# Overcoming the Impact of *Students for Fair Admission v Harvard* to Build a More Representative Health Care Workforce: Perspectives from *Ending Unequal Treatment*

**DOI:** 10.1111/1468-0009.12718

**Published:** 2024-10-03

**Authors:** VINCENT GUILAMO‐RAMOS, MARCO THIMM‐KAISER, ADAM BENZEKRI, RUTH S. SHIM, FRANCIS K. AMANKWAH, SARA ROSENBAUM

**Affiliations:** ^1^ Institute for Policy Solutions, School of Nursing Johns Hopkins University; ^2^ Center for Latino Adolescent and Family Health Johns Hopkins University; ^3^ US Presidential Advisory Council on HIV/AIDS; ^4^ Mailman School of Public Health Columbia University; ^5^ Steinhardt School of Culture, Education, and Human Development New York University; ^6^ University of California – Davis Health; ^7^ National Academies of Sciences, Engineering, and Medicine; ^8^ Milken Institute School of Public Health George Washington University

**Keywords:** Ending Unequal Treatment, health equity, health care workforce

## Abstract

**Policy Points**

- In a recently commissioned report on solutions for eliminating racial and ethnic health care inequities entitled *Ending Unequal Treatment*, the National Academies of Sciences, Engineering, and Medicine found a health workforce that is representative of the communities it serves is essential for health care equity.
- The Supreme Court decision to ban race‐conscious admission constraints pathways toward health workforce representativeness and equity.
- This paper draws on the National Academies report's findings that health care workforce representativeness improves care quality, population health, and equity to discuss policy and programmatic options for various participants to promote health workforce representativeness in the context of race‐conscious admissions bans.

The united states supreme court's decision in *Students for Fair Admissions, Inc., v President and Fellows of Harvard College* (*SFFA*) has had a major impact on university admissions practices, not only at the undergraduate level (the focus of the legal challenge) but at the graduate level as well, including for health profession programs.^1^ Indeed, health professional education has been a specific target of the attack on affirmative action since the seminal 1978 decision in *Regents of the University of California v Bakke*,^2^ which barred the use of medical school minority admissions quotas while also recognizing diversity in higher education as a compelling governmental interest that justified other strategies, including use of statistical data, to achieve a diverse student body. By expressing skepticism of racial and ethnic diversity in educational admissions as a compelling educational goal in its own right, *SFFA* imposes legal barriers that will likely end race‐conscious admissions in practice for the foreseeable future while at the same time leaving the door open to individualized, limited consideration of applicants’ experience based on their race and ethnicity.

The National Academies of Sciences, Engineering, and Medicine's recent report *Ending Unequal Treatment: Strategies to Achieve Equitable Health Care and Optimal Health for All*, released in the wake of *SFFA*, concludes that the evidence strongly shows the value of a diverse health care workforce in achieving a more equitable health care system.^3^ In the context of the *Ending Unequal Treatment* findings, this article discusses the imperative of a representative health care workforce as a core element of any strategy for overcoming the persistence of health and health care inequities as well as options for future programmatic and policy action in the face of race‐conscious admissions bans.

## The History of Inequity in Health Care

For many decades, racial and ethnic health inequities have been recognized as a defining characteristic of the US health system. In 1984, Secretary Margaret M. Heckler of the US Department of Health and Human Services established the Task Force on Black and Minority Health, presenting a landmark report acknowledging the deleterious effects of structural racism on health inequities among US communities of color.^4^ In response to Secretary Heckler's report, the Centers for Disease Prevention and Control founded its Office of Minority Health and Health Equity in 1988.^5^ Racism and implicit bias as key determinants of health and health care inequities received renewed attention in 2003 when the National Academies published its groundbreaking *Unequal Treatment* report.^6^ In June 2024, the National Academies released their updated consensus report *Ending Unequal Treatment*, which represents a 20‐year reevaluation of progress since the original 2003 report.^3^

In spite of this long‐standing recognition of the harmful impacts of structural racism and other structural and social determinants of health, the report found little progress in reducing health and health care inequities in the United States over the past two decades.^3^ Given that population‐level health inequities are largely driven by complex structural determinants of health—including oppression and structural racism—that operate both inside and outside of health care services and systems, the identification of promising programmatic and policy actions aimed at improving care across all patients and institutions was a key priority.^3^ One such opportunity for policy intervention is legislation, regulation, and financing that pertains to the US health care workforce in which racially and ethnically minoritized providers remain vastly underrepresented despite evidence that workforce representativeness is associated with improved health outcomes of racially and ethnically minoritized persons and communities who are disproportionately affected by health and health care inequities.^3^

## The Importance of a Representative Health Care Workforce

Extensive research has documented the benefits associated with a diverse health care workforce. For example, even in the absence of a fully population‐representative health care workforce, increasing racial and ethnic diversity among health care professionals ensures greater exposure of the dominant group of providers to minoritized peers. These interactions provide opportunities for counterstereotyping, mitigating prevalent implicit bias around racial and ethnic hierarchies, and infusing new perspectives and differing forms of lived experience that can improve the quality of health care practice, research, and teaching.^7^, ^8^, ^9^ *Ending Unequal Treatment* documents particularly strong evidence for the beneficial effects of racial and ethnic patient–provider concordance (that is, better outcomes for racially and ethnically minoritized patients receiving care from racially and ethnically concordant health care providers).^3^ Based on this strong empirical evidence, patient–provider concordance is a particularly important foundation for establishing the significance of a representative health care workforce.

The overall evidence reviewed in the *Ending Unequal Treatment* report shows that racially and ethnically minoritized patients who receive care from racially and ethnically concordant health care providers generally have better health outcomes.^3^ These conclusions rest on a substantial body of research the methodological rigor, operationalization of concordance, and health outcomes under study of which vary widely,^10^, ^11^ leading to mixed results. Nevertheless, several large and methodologically rigorous studies point to the benefits of patient–provider concordance. For example, a randomized controlled trial among 1,300 Black men assigned to receiving care from a racially concordant or nonconcordant health care provider found improved preventive service uptake for concordant patient–provider dyads.^12^ When extrapolating the attained improvement in preventive care to the US population, the researchers estimated that a 19% reduction in the cardiovascular mortality gap between Black and White men could be achieved.^12^ An analysis of nearly two million hospital births in Florida between 1992 and 2015 found a newborn mortality rate that was close to three times higher among Black newborns than among White newborns.^13^ Notably, the Black–White mortality gap was more than halved when Black providers delivered care to Black newborn children. There was no difference in outcomes for White newborns, regardless of provider race and ethnicity, illustrating the outsized importance of health workforce representativeness for racially and ethnically marginalized communities experiencing preexisting health inequities.

Evidence also suggests that the benefits of a health care workforce that represents the communities it serves extend to the population level. Snyder and colleagues found that counties with higher representation of Black health professionals among primary care providers had a higher life expectancy and lower all‐cause mortality among Black county residents.^14^ In addition, as representation of Black health professionals increased, the observed Black–White inequity in all‐cause mortality in the county decreased.^14^ Similarly, Frakes and Gruber found that an increase in the proportion of Black providers in the US Military Health System was associated with a significant 15% decrease in all‐cause mortality among Black patients with chronic conditions.^15^

Although the majority of existing research has evaluated the role of health care provider representativeness and concordance for Black patients and providers, the body of evidence substantiating benefits for other racial and ethnic groups is growing. For example, racial and ethnic patient–provider concordance is associated with greater engagement in preventive care, greater care seeking for new health problems, and better continuity of care for Latino, Asian, and Black individuals.^16^ An evaluation of health care expenditure data also suggests better care outcomes when patients and providers share a racial or ethnic background. Specifically, Latino and Asian patients who saw concordant health care providers had a lower risk of emergency department visits, and Latino patients also recorded fewer hospitalizations.^17^ In addition, Latino, Asian, and Black patients with concordant providers accrued lower overall health care expenditures.^17^

## Insights Into Why Patient–Provider Concordance Works

The benefits of patient–provider racial and ethnic concordance justify increasing health care workforce representativeness as a priority health improvement strategy for underrepresented racially and ethnically minoritized populations experiencing health inequities. However, patient–provider racial and ethnic concordance cannot and should not be a singular solution. Patient–provider concordance aside, health care professionals need skills and techniques to build strong relationships with all patients, regardless of their race, ethnicity, or needs. Understanding the ways in which patient–provider concordance improves care experiences and outcomes thus becomes a valuable means of identifying strategies that may be generalizable to the entire health care workforce.

The existing literature on these mechanisms remains inconclusive. Nevertheless, it can provide important insights despite gaps. The largest body of work has focused on the interpersonal aspects of patient–provider interactions, such as communication, shared decision making, discrimination, and bias, along with subsequent patient‐level outcomes, such as care satisfaction, perceived quality of care, trust, and adherence to treatment and recommendations.^18^, ^19^, ^20^ This research provides some, albeit mixed, evidence for these factors playing a mediating role in some care contexts and for some health outcomes.^18^, ^19^ However, there is also research documenting the benefits of patient–provider concordance in care contexts in which patient‐level perceptions of interactions with providers and care processes are unlikely to be the primary operating mechanisms. For example, improved outcomes for Black newborns delivered by Black health care providers are difficult to explain by patient–provider communication, shared decision making, or trust.^13^ Instead, these data may reflect tangible differences in care quality that are most likely the result of implicit bias and structural racism impacting providers’ decision making. Studies have shown differences in care decisions and counseling on the part of providers in concordant vs. discordant patient–provider dyads. For example, research has found patient–provider concordance to be associated with more appropriate antibiotic prescriptions and differences in weight‐related counseling among patients who have obesity.^21^, ^22^

Taken together, the available literature suggests that the mechanisms of patient–provider concordance include both interpersonal pathways and pathways related to provider care decisions and delivery. Unfortunately, the substantive evidence remains too scarce to directly inform the development of targeted programming or policy. Therefore, advancing the understanding of the mechanisms of patient–provider concordance represents an important and potentially impactful area of research.

## Health Care Workforce Representation in the Context of Recent Supreme Court Decisions

The *Ending Unequal Treatment* report explicitly discusses the issue of health care workforce representativeness in relation to the goal of health care equity.^3^, ^23^ Specifically, the report finds that a health care workforce that is representative of the diverse communities it serves provides an important cornerstone of a more equitable health care system—and further that previous efforts to advance this goal have largely failed. In addition, the report finds evidence that the Supreme Court's decision in *SFFA* that bans race‐conscious admissions in higher education, including health professional education, imposes constraints on effective pathways toward health workforce representativeness and equity.^1^ Therefore, the report concludes that “a diverse health and science workforce, representative of the communities that it serves, is essential to health care equity. The nation has made little progress addressing this goal. Recent court decisions concerning diversity, equity and inclusion serve to further limit progress in achieving a diverse workforce.”^3^ For example, a recent study found a nearly 5% decline in underrepresented racial and ethnically minoritized students in public medical schools in states that had implemented bans on race‐conscious admissions, whereas control states without such bans recorded an 0.7% increase over the same time period.^24^ Furthermore, the Massachusetts Institute of Technology reported that the share of Latino, Black, and Native American or Pacific Islander students in the first incoming freshman class following *SFFA* fell precipitously to only 16%, compared with approximately 25% in the years prior.^25^ It is also noteworthy that the restrictions on race‐conscious admissions in the wake of *SFFA* and the associated loss of diversity and representativeness in US higher education coincide with the threat of a broad backlash against diversity, equity, and inclusion (DEI) programming in higher education, which is characterized by a growing number of state legislative initiatives to prohibit DEI offices or staff, mandatory DEI training, the use of DEI statements in hiring or promotion, and other means of achieving diversity.^26^

The Supreme Court heard extensive arguments emphasizing the measurable value of racial and ethnic diversity in higher education as a means of enriching the quality of education and ensuring equality of opportunities.^1^ These arguments were bolstered by ample scientific evidence.^27^ At the same time, however, the unique importance of diversity in health professional education specifically received less attention, in particular the role of health care workforce representativeness in improving care quality, population health, and health equity—a common good that depends on a diverse body of health professional trainees across health care disciplines.^28^, ^29^, ^30^, ^31^, ^32^, ^33^, ^34^

## Options for Programmatic and Policy Action in the Context of Race‐Conscious Admission Bans

The compelling body of evidence substantiates the need for greater health care workforce representativeness as a key element of a comprehensive strategy to build a more equitable—and more sustainable—US health system. Although a ban on race‐conscious admissions policies may impose a major restriction on an especially important key strategy, various participants, including institutions of higher education, educational accrediting organizations, health professional organizations, institutional health system leaders, policymakers, and government agencies nonetheless continue to have a range of levers at their disposal that remain viable and legal to shape the recruitment, training, and practice of health professionals. *SFFA* heavily restricts the use of race consciousness but leaves other important policy and programmatic levers untouched. We present several promising programmatic and policy options that include potentially exempting health professions education from the limitations imposed by the Supreme Court, as well as other federal and state policy strategies, targeted program funding, decisions by institutions of higher education, and faculty diversity.

### Exempting Health Professions Education Programs From SFFA Limits

The Supreme Court opinion banning race‐conscious admissions in US higher education hinted at a potential exemption for the military academies given their potentially “distinct interests” in a racially and ethnically representative student body.^1^ Based on the evidence, the same could be said for health care. Indeed, US civil rights law, which contains a specific prohibition against discrimination in health care, added by Section 1557 of the Affordable Care Act, underscores the express federal interest in explicit strategies that can address racial and ethnic health care inequities.^3^ Although any continuation of race‐conscious admission in health professional schools will almost certainly be contested in court, we believe that this is a battle worth having given the strength of the evidence for creating such an exemption.^35^ Scholars have already put forward well‐founded legal arguments for a compelling interest in racial and ethnic diversity among students admitted to health professional education (see, for example, Cole and Curfman).^36^ A clear and supportive government stance in favor of such an exemption is warranted in our view.

### Federal Policy Action

Affirmative action in the United States was first introduced as a federal policy concept through an executive orders issued by President John F. Kennedy in 1961.^37^ Title VI of the 1964 Civil Rights Act codified the obligation of entities receiving federal financial assistance not to discriminate on the basis of race, color, or national origin; health care was a major focus of the 1964 Act.^38^ Over decades, the breadth and scope of federal nondiscrimination laws has expanded, culminating in the enactment of Section 1557 of the Affordable Care Act, which modernizes, strengthens, and expands the concept of nondiscrimination in health care, including discrimination based on the factors addressed by Title VI. From the earliest days of nondiscrimination law, higher education was a focus, as was health care delivery itself. In the wake of *SFFA*, the question is how this singular commitment to racial justice in health care should manifest itself in health profession education policy given the degree to which health workforce diversity can so significantly affect equity in health care and health. Whether health care, like the military, might represent a special case and, if so, what diversity efforts barred in undergraduate settings nonetheless might be permissible in health care education and training programs has emerged as a subject of high interest among legal scholars and health policy leaders. Putting aside legal frontiers yet to be crossed, the question becomes which policy levers might prove fruitful in achieving diversity.

Furthermore, the numerous federal government agencies invest in health professional students from underserved and racially or ethnically minoritized communities. These programs include, for example, the Health Resources and Services Administration's Nursing Workforce Diversity Program and the National Institutes of Health's F31, R36, F99/K00, and K99/R00 fellowship and grant programs to support underrepresented early‐career health researchers.^39^, ^40^ Expansion of these programmatic investments represents an important lever to support opportunities for racially and ethnically minoritized entrants into the health professions and the research workforce.

### State‐Level Legislation

Research that evaluated the impact of state‐level legislative changes in seven US states designed to promote recruitment of underrepresented minoritized entrants to careers in nursing in comparison with seven neighboring control states without such legislation suggests that targeted legislation is promising, but likely insufficient by itself, for increasing the diversity and representativeness of the health care workforce.^41^ In particular, legislation that expressed support and encouragement for underrepresented minoritized professionals to enter the health workforce, legislation that tied reimbursement for health services to efforts for workforce diversification at the institutional level, and legislation that appropriated funding for programs (e.g., grants, loans, scholarships, etc.) directly supporting underrepresented minoritized entrants into the health professions were most likely to show greater racial and ethnic workforce diversity relative to the prelegislation period and relative to control states.^41^

### Targeted Programmatic Funding

Funding decisions about investments in different types of health care workforce development have shown promise for increasing workforce diversity and representativeness.^42^

#### Increasing Residency or Clinical Training Program Capacity

For many health professions, residency and/or clinical training program capacities are below the demand for new health care workers.^43^, ^44^ For example, the limited availability of clinical placements represents a constraint on nursing schools, preventing them from admitting qualified applicants.^45^ Similarly, there are insufficient supervised training placements and residency slots for behavioral health professionals, including adult and child psychiatrists, clinical psychologists, social workers, counselors, and mental health nurses, available to meet mental health workforce needs.^46^, ^47^, ^48^ In the absence of race‐conscious admissions policies, the available training program and residency placements are less likely to be allocated to underrepresented minoritized students and trainees because of structural biases in how meritocracy is presently operationalized, such that it more closely reflects access to resources than it does talent or potential for professional growth and success.^28^, ^30^ Federal, state, and local funding streams can offset the organizational costs associated with creating more residency and training program capacity. For example, the Centers for Medicare and Medicaid Services’ graduate nurse education demonstration, which provided funding for clinical training of advanced practice nurses similar to existing graduate medical education funding, succeeded in increasing nurse practitioner enrollments and graduations, including in primary care specializations.^49^ Inadequate residency and training program capacities often represent an acute bottleneck impacting health professionals already in training. Therefore, expansion of available programs represents an investment with potentially immediate impacts. However, research suggests that investments to increase residency or training program capacity only improve workforce diversity and representativeness if the funding mechanisms stipulate specific requirements for increased residency/training program cohort diversity.^42^

#### Loan Repayment Programs

Loan repayment programs, often linked to a service commitment in a defined geographic area or care setting, can reduce the financial burden associated with health professional education, which often disproportionately affects underrepresented minority students and early‐career health care workers.^50^, ^51^ Importantly, research suggests that loan repayment programs for early‐career health professionals are most impactful for improving workforce diversity and representativeness if they are specifically targeted to underrepresented minoritized entrants.^42^

#### Service‐Contingent Scholarship Programs

Service‐contingent scholarship programs serve to reduce financial barriers to entry of health professional education and to provide a return for federal, state, or local funders by eliciting student commitments for postgraduation practice in a specific geography in primary care or a specific specialty.^52^ Adequate options for financial support have been shown to be particularly important for recruiting underrepresented minoritized students into health professional education.^52^ Importantly, research suggests that scholarship programs for early‐career health professionals are most impactful for improving workforce diversity and representativeness if they are designed to specifically appeal to and reach underrepresented minoritized students.^42^

#### Pathway Programs

Health professional pathway programs are designed to support high school, college, or postbaccalaureate students in acquiring the requisite experiences and skills for entry into health professional training.^42^, ^53^ Evidence suggests that pathway programs are likely associated with increased opportunities for underrepresented minoritized students to enter the health professions.^54^ Notably, pathway programs specifically designed to recruit and support students who are underrepresented in the health professions have the strongest evidence of effectiveness for improving workforce diversity of the four highlighted program types.^42^ These programs can include support for partnerships between health professional schools and high schools in underserved zip codes and funding for entrance exam preparatory services (e.g., preparation for the medical college admission test [MCAT], nursing preadmission exam [PAX], etc.) at Historically Black Colleges and Universities, Hispanic‐Serving Institutions, Tribal Colleges and Universities, and undergraduate schools and community colleges with high socioeconomic, racial, and ethnic diversity.^30^, ^36^

### Decisions by Institutions of Higher Education

Given that, ultimately, admissions decisions rest with the institutions of higher education that are delivering health professional education, they will continue to hold a large amount of agency in determining the diversity and representativeness of future health professional trainees—including after the restrictions on race‐conscious admissions imposed by *SFFA*. Leading up to and following the *SFFA* decision, there have been robust discussions in the scientific and legal literature of the opportunities and responsibilities for institutions of higher education to—within the new legal boundaries—make decisions that prevent new barriers for underrepresented and racially or ethnically minoritized entrants into the health professions.^28^, ^29^, ^30^, ^31^, ^32^, ^33^, ^34^, ^35^, ^36^ These include an embrace, scaleup, and funding of the pathway programs and institutional partnerships described in the previous sections but also changes to long‐standing institutional practices, such as ending legacy admissions, which under the Supreme Court's “zero‐sum” rationale on university admissions, clearly disadvantage historically underrepresented students.^29^ Furthermore, institutions of higher education ought to make use of other elements of holistic review that are explicitly permitted under *SFFA* to offset the structural barriers faced by racially or ethnically minoritized applicants. Strong and steadfast support for these initiatives from educational accreditation bodies, such as the Liaison Committee on Medical Education, Accreditation Council for Graduate Medical Education, or the Commission on Collegiate Nursing Education, and school alumni in influential health professional organizations, such as the American Medical Association or the American Nurses Association, will be essential.

### Diverse Faculty

Finally, efforts to increase racial and ethnic representativeness need to go beyond the student body and extend to greater diversity among health professional school faculty.^43^ Faculty diversity has proven to be important for increasing institutional diversity in higher education, and a lack of faculty diversity represents a barrier to the recruitment of a diverse and representative student body.^55^, ^56^ Research suggests that increased faculty diversity is fundamental for shaping an organizational culture that is welcoming to underrepresented minoritized students and that greater representation of underrepresented minoritized faculty on admissions committees was associated with increased diversity of the matriculated student body.^42^

## Conclusions

*Ending Unequal Treatment* finds that increasing the racial and ethnic representativeness of the US health workforce is indispensable for building an equitable and sustainable US health system—a pressing national priority. Despite recent federal race‐conscious admissions bans, a range of promising programs and strategies to promote the recruitment and training of underrepresented minoritized health professionals remain available. However, in the absence of broad recognition and commitment to the crucial importance of a representative health care workforce by all interested parties, including institutions of higher education, educational accrediting organizations, health professional organizations, and institutional health system leaders—and absent adequate and sustained regulatory and fiscal support from policymakers and government agencies—their adoption and scaleup is all but guaranteed to fall short of meaningful progress toward health workforce representativeness. A failure to act on these policy priorities would hurt all Americans and most of the communities already affected by the long‐standing racial and ethnic health inequities that remain characteristic of the United States.

## Conflict of Interest Disclosures

V.G.‐R., R.S.S., and S.R. served as members of the National Academies of Sciences, Engineering, and Medicine's (NASEM) Committee on Unequal Treatment Revisited, which developed the *Ending Unequal Treatment* report. F.K.A. was the responsible staff officer at NASEM. M.T.‐K. and A.B. have nothing to disclose.
